# Labeling and measuring stressed mitochondria using a PINK1-based ratiometric fluorescent sensor

**DOI:** 10.1016/j.jbc.2021.101279

**Published:** 2021-10-05

**Authors:** Rie Uesugi, Shunsuke Ishii, Akira Matsuura, Eisuke Itakura

**Affiliations:** 1 Department of Biology, Graduate School of Science and Engineering, Chiba University, Chiba, Japan; 2 Department of Biology, Graduate School of Science, Chiba University, Chiba, Japan

**Keywords:** mitochondria, PTEN-induced putative kinase 1 (PINK1), mitochondrial stress, mitochondrial membrane potential, mitochondrial sensor, Parkin, CCCP, carbonyl cyanide m-chlorophenyl hydrazone, DAPI, 4′,6-diamidino-2-phenylindole, ER, endoplasmic reticulum, KD, kinase dead, mito-Pain, mitochondrial PINK1 accumulation index, mito-Pain F, mitochondrial PINK1 accumulation index (containing full-length PINK1), mito-Pain T, truncated mito-Pain, MTR, MitoTracker Red, MTS, mitochondrial targeting sequence, OMM, outer mitochondrial membrane, PINK1, PTEN-induced putative kinase 1, PMA, phorbol 12-myristate 13-acetate, ROS, reactive oxygen species, RTK, receptor tyrosine kinase, TERT, telomerase reverse transcriptase, TMD, transmembrane domain

## Abstract

Mitochondria are essential organelles that carry out a number of pivotal metabolic processes and maintain cellular homeostasis. Mitochondrial dysfunction caused by various stresses is associated with many diseases such as type 2 diabetes, obesity, cancer, heart failure, neurodegenerative disorders, and aging. Therefore, it is important to understand the stimuli that induce mitochondrial stress. However, broad analysis of mitochondrial stress has not been carried out to date. Here, we present a set of fluorescent tools, called mito-Pain (mitochondrial PINK1 accumulation index), which enable the labeling of stressed mitochondria. Mito-Pain uses PTEN-induced putative kinase 1 (PINK1) stabilization on mitochondria and quantifies mitochondrial stress levels by comparison with PINK1-GFP, which is stabilized under mitochondrial stress, and RFP-Omp25, which is constitutively localized on mitochondria. To identify compounds that induce mitochondrial stress, we screened a library of 3374 compounds using mito-Pain and identified 57 compounds as mitochondrial stress inducers. Furthermore, we classified each compound into several categories based on mitochondrial response: depolarization, mitochondrial morphology, or Parkin recruitment. Parkin recruitment to mitochondria was often associated with mitochondrial depolarization and aggregation, suggesting that Parkin is recruited to heavily damaged mitochondria. In addition, many of the compounds led to various mitochondrial morphological changes, including fragmentation, aggregation, elongation, and swelling, with or without Parkin recruitment or mitochondrial depolarization. We also found that several compounds induced an ectopic response of Parkin, leading to the formation of cytosolic puncta dependent on PINK1. Thus, mito-Pain enables the detection of stressed mitochondria under a wide variety of conditions and provides insights into mitochondrial quality control systems.

Mitochondria are complex organelles that play a variety of roles in various metabolic processes, including energy production, lipid synthesis, and protein translation, and as dynamic organelles, they frequently exhibit changes in shape, size, and distribution in response to various stimuli. These proper functions of mitochondria are essential for cell survival (1). ATP production through the respiration chain is a key activity in eukaryotes, but respiration in mitochondria generates reactive oxygen species (ROS) (2, 3), which induce oxidative stress, leading to damage of mitochondrial proteins, DNA, and lipids (4, 5, 6). Misfolding of mitochondrial proteins and defects in protein transport through mitochondrial translocons injure mitochondria as well (7, 8). The effects of damaged mitochondria (*e.g.*, generating ROS) exacerbate DNA mutagenesis and lead to several deleterious outcomes, such as aging and neoplastic transformation. To prevent these negative effects, quality control systems remove mitochondrial damaged proteins through the action of mitochondrial proteases, which degrade mitochondrial proteins, or by mitophagy, which degrades damaged mitochondria by autophagy (8, 9, 10). Therefore, defects in mitochondrial quality control systems increase the number of damaged mitochondria, which results in several diseases, such as Parkinson’s disease (11).

Mitochondrial depolarization is associated with mitochondrial stress. Because several fluorescent dyes, including tetramethylrhodamine methyl ester and MitoTracker, specifically stain polarized mitochondria in cells, measurement of the fluorescence intensity using these reagents enables the detection of mitochondrial depolarization (12, 13). JC-1, a ratiometric dye that stains polarized mitochondria red and depolarized mitochondria green, is a sensitive indicator of mitochondrial depolarization. However, most of them are not suitable for use with fixation (14). Another problem is that, all the reagents measure only mitochondrial stress accompanied with mitochondrial depolarization. To understand mitochondrial stress, it is important to quantitatively detect a broad range of mitochondrial stress types in addition to depolarization. Mitochondria are complex organelles in which most mitochondrial proteins are encoded in the nuclear DNA, and highly dynamic machines drive and contribute to mitochondrial translocases to import mitochondrial proteins (15). However, disturbances of mitochondrial homeostasis by mitochondrial stress cause a reduction in the protein import activity. Hence, we hypothesized that measurement of mitochondrial import activity can be an indicator of mitochondrial stress.

PTEN-induced putative kinase 1 (PINK1) is a mitochondrial protein identified as a responsible gene in Parkinson’s disease (16, 17). PINK1 is a Ser/Thr kinase that undergoes autophosphorylation and phosphorylates Parkin and ubiquitin. PINK1–Parkin–mediated mitophagy has been well characterized, and Parkin activated by PINK1 ubiquitinates outer mitochondrial proteins that are targeted for mitophagy, leading to the degradation of damaged mitochondria (18, 19, 20). PINK1 is the critical factor in mitophagy because PINK1 functions at the initial mitophagy step to distinguish healthy and stressed mitochondria. For healthy mitochondria, translated PINK1 is transported into mitochondria through the Tom complex on the outer mitochondrial membrane (OMM) and the Tim23 complex on the inner mitochondrial membrane by a mitochondrial targeting sequence (MTS). After passing through the Tim–Tom complex, a mitochondrial protease cleaves the N-terminal MTS of PINK1. Then, PARL, an inner mitochondrial membrane–resident protease, further cleaves PINK1 within the transmembrane domain (TMD). This truncated PINK1 is retrotranslocated into the cytosol and degraded by the proteasome (21, 22, 23). In stressed mitochondria (*e.g.*, depolarized mitochondria), the inactivated TIM23 complex does not allow PINK1 passage; therefore, PINK1 is not cleaved by PARL. Noncleaved PINK1 accumulates on the OMM, where it is stabilized by autophosphorylation and multimerization, thereby recruiting and then phosphorylating Parkin, which subsequently ubiquitinates the mitochondrion for mitophagy (23, 24, 25).

PINK1 is a mitochondrial quality control factor in addition to inducing Parkin-mediated mitophagy upon mitochondrial depolarization. Besides import inhibition caused by depolarization, misfolding proteins in the mitochondrial matrix or disturbance of mitochondrial calcium density also leads to PINK1 recruitment to mitochondria (10, 26). So, mitochondrial PINK1 can be used as an indicator of mitochondrial stresses. In addition, considering that the expression of Parkin is restricted to some tissues, such as nerves and the liver, ubiquitous expression of PINK1 may explain that PINK1 has a Parkin-independent role in mitochondrial quality control (27, 28). Indeed, HeLa cells express endogenous PINK1, which is recruited to depolarized mitochondria without the expression of Parkin (21), suggesting that PINK1 might have roles in the mitochondrial quality control system independent of Parkin. Therefore, a broad exploration of the PINK1-mediated mitochondrial quality control system will contribute to the elucidation of the molecular mechanism of mitochondrial quality control.

To address the challenge posed by measuring mitochondrial stress, we generated a novel ratiometric fluorescent sensor using PINK1 that quantitively measures various mitochondrial stresses. We screened 3374 compounds using the new screening strategy and identified various types of mitochondrial stress inducers, including depolarization-independent and Parkin-independent mitochondrial stress. This novel tool is cost-effective and fixable, and it can be used to measure various mitochondrial stresses in culture cells quantitatively.

## Results

### Development of a novel fluorescent sensor for quantification of mitochondrial stress

**Figure 1:**
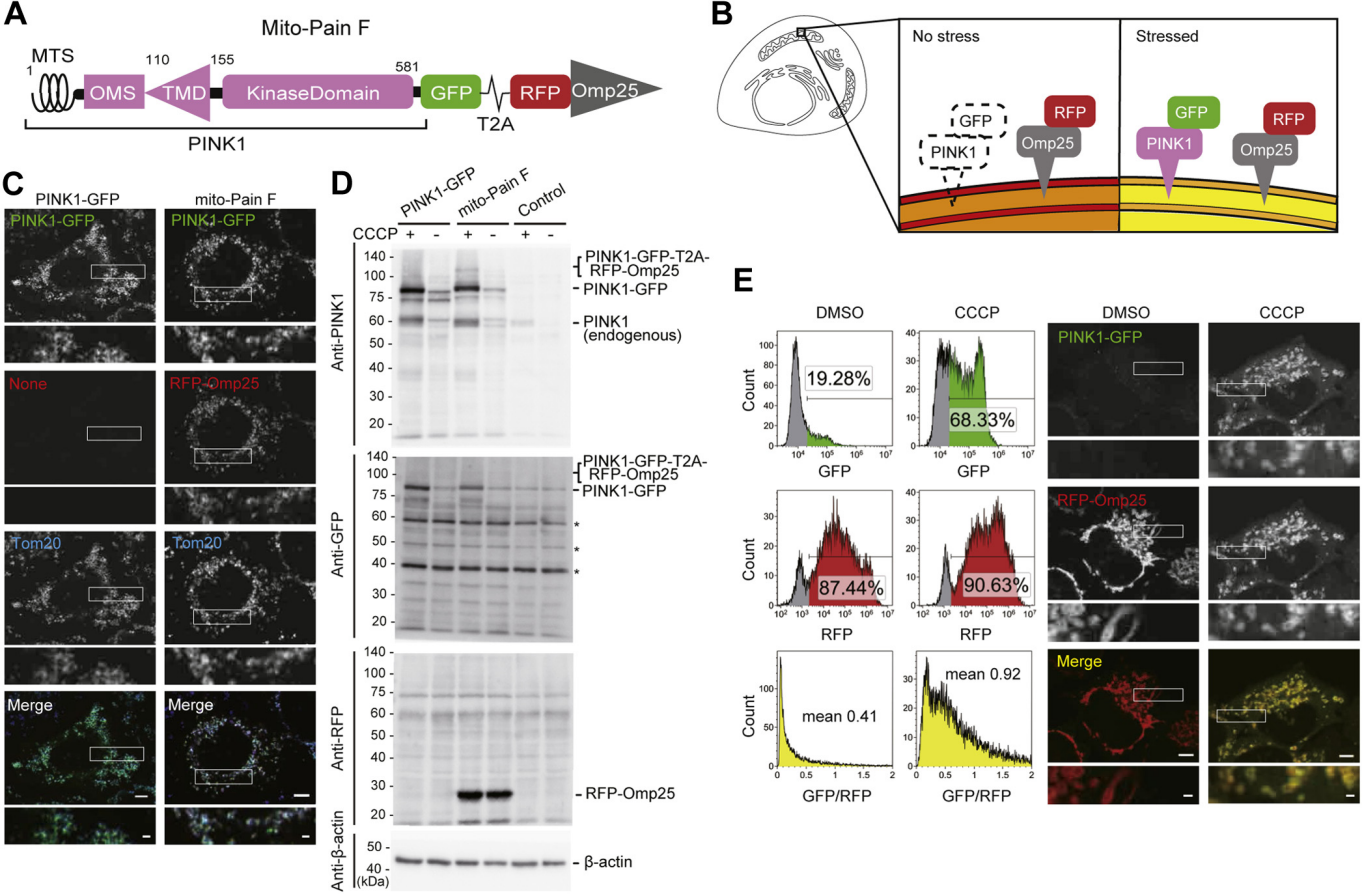
**Development of a novel sensor to detect mitochondrial stress.***A*, schematic representation of mito-Pain F. *Numbers* above the construct indicate amino acid residues. *B*, a schematic diagram of the measurement of mitochondrial stress by mito-Pain. *C*, the T2A peptide sequence did not interfere with the localization of PINK1-GFP. HeLa cells stably expressing mito-Pain F or PINK1-GFP were cultured with CCCP for 24 h before fixation. The cells were stained with an antibody against Tom20 and observed by fluorescence microscopy. The scale bar represents 5 μm. The inset scale bar represents 1 μm. *D*, mito-Pain F is efficiently cleaved at the T2A site. HeLa cells and HeLa cells expressing mito-Pain F or PINK1-GFP were treated with CCCP or DMSO for 24 h and analyzed by immunoblotting using antibodies against PINK1, GFP, and RFP. *Asterisks* indicate nonspecific bands. *E*, PINK1-GFP, but not RFP-Omp25, of mito-Pain F was significantly increased under mitochondrial depolarization. HeLa cells stably expressing mito-Pain F were cultured with CCCP or DMSO for 24 h and then analyzed by flow cytometry. Cells for fluorescence microscopy were subjected to the same treatment and then fixed. The data represent the mean (*left bottom panel*: GFP-RFP fluorescence ratio) (n = 9). The main scale bar represents 5 μm. The inset scale bar represents 1 μm. CCCP, carbonyl cyanide m-chlorophenyl hydrazone; DMSO, dimethyl sulfoxide; mito-Pain, mitochondrial PINK1 accumulation index; mito-Pain F, mitochondrial PINK1 accumulation index (containing full-length PINK1); MTS, mitochondrial targeting sequence; OMS, outer mitochondrial membrane localization signal; PINK1, PTEN-induced putative kinase 1; TMD, transmembrane domain.

To quantify mitochondrial stress, we generated a plasmid-based ratiometric fluorescent sensor constructed with PINK1-GFP, which is stabilized on mitochondria in a stress-dependent manner. PINK1-GFP was linked with the T2A “self-cleaving” peptide sequence (29, 30) and fused with RFP and the TMD of Omp25 (31), which is a tail-anchored protein that is inserted into the OMM in a stress-independent manner to compensate for its expression level. The resultant plasmid (full-length PINK1-GFP-T2A-RFP-Omp25TMD) is referred to as the mitochondrial PINK1 accumulation index (containing full-length PINK1) (mito-Pain F) (Fig. 1*A*). During translation, PINK1-GFP-T2A-RFP-Omp25TMD is cleaved at the T2A sequence, and the former region generates PINK1-GFP, which is transported to mitochondria by the MTS in the N terminus of PINK1. The latter region constitutes RFP-Omp25. In healthy mitochondria, PINK1-GFP is imported into mitochondria and then rapidly undergoes cleavage by a mitochondrial inner-membrane protease and is then retrotranslocated into the cytosol. Cytosolic cleaved PINK1-GFP is immediately degraded by the proteasome. In contrast, stressed mitochondria (*e.g.*, depolarization) do not allow the translocation of PINK1-GFP through the inner membrane complex. Therefore, PINK1-GFP is localized and stabilized on the OMM (Fig. 1*B*). However, RFP-Omp25, the internal control of expression level, is always localized on the OMM, which enables quantification by calculating the GFP-RFP signal ratio of mito-Pain F under mitochondrial stress.

To assess whether mito-Pain F is capable of indicating mitochondrial stress, we generated HeLa cells stably expressing mito-Pain F using a lentivirus system. In the cells expressing PINK1-GFP alone, GFP colocalized with the outer mitochondrial protein TOM20 upon mitochondrial depolarization induced by treatment with carbonyl cyanide m-chlorophenyl hydrazone (CCCP), as expected (Fig. 1*C*). Cells expressing mito-Pain F also showed colocalization of GFP and RFP-OMP25 with Tom20. An immunoblot analysis showed that mito-Pain F expressed PINK1-GFP and RFP-Omp25 separately, and T2A cleavage was efficient, with a rate higher than 99.3%, and exogenous PINK1 was expressed several-fold more than endogenous PINK1 (Fig. 1*D*). These data indicate that the addition of the T2A peptide did not affect the localization and the stability of PINK1-GFP. PINK1-GFP was very low under basal conditions (Fig. 1, *D* and *E*). In contrast, induction of mitochondrial depolarization markedly increased GFP fluorescence intensity, and the GFP-RFP ratio under stressed conditions was 2.2-fold higher as that under basal conditions. We also confirmed that CCCP-induced PINK1-GFP in cells expressing mito-Pain F colocalized with RFP-Omp25 (Fig. 1*E*, right panel). These results suggest that mito-Pain F indicates mitochondrial stress.

### Kinase domain–deleted mito-Pain T

**Figure 2:**
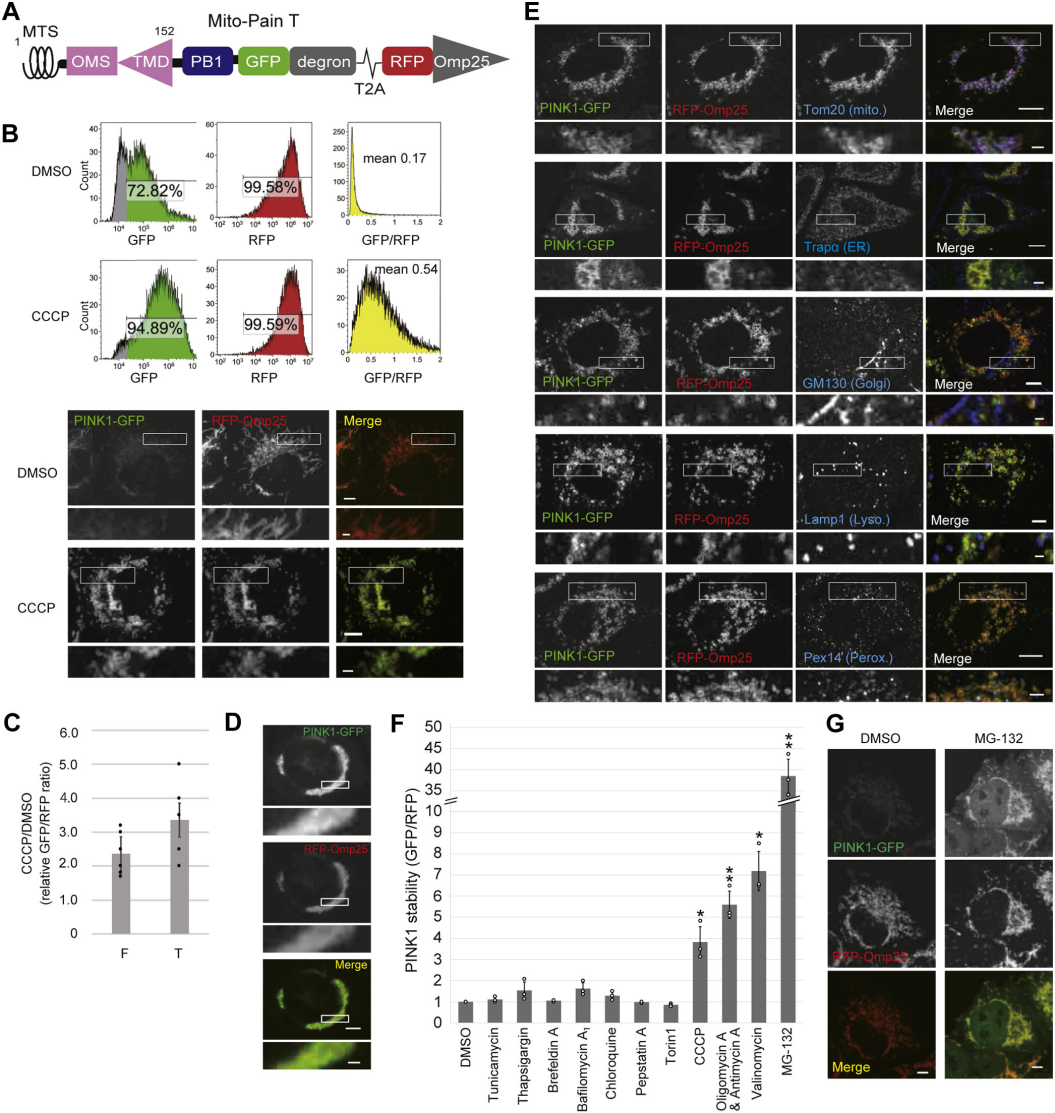
**Improvement of mito-Pain by removing the PINK1 kinase domain.***A*, schematic representation of mito-Pain T (PINK1 truncated). *Numbers* above the construct indicate amino acid residues of each protein (the PB1 domain from PKCζ and the degron domain from Rpn4.) *B*, mito-Pain T showed a much higher signal ratio than mito-Pain F. Polyclonal HeLa cells stably expressing mito-Pain T were cultured with CCCP or DMSO for 24 h and then analyzed by flow cytometry. Cells for fluorescence microscopy were subjected to the same treatment and then fixed. The data represent the mean (*right panel*: GFP-RFP fluorescence ratio) (n = 7). The scale bar represents 5 μm. The inset scale bar represents 1 μm. *C*, graph showing the results of quantification of GFP-RFP fluorescence ratios (n = 9 for mito-Pain F and n = 7 for mito-Pain T). The data represent the mean ± SD. *D*, high expression of mito-Pain T–induced mitochondrial aggregation. HeLa cells highly expressing mito-Pain T were cultured with CCCP for 24 h before fixation. The scale bar represents 5 μm. The inset scale bar represents 1 μm. *E*, the GFP signal of mito-Pain T mainly localized to mitochondria under mitochondrial depolarization. HeLa cells expressing mito-Pain T were cultured with CCCP for 24 h before fixation. The cells were stained with antibodies against Tom20, Trapα, GM130, Lamp1, or Pex14 and observed by fluorescence microscopy. The scale bar represents 5 μm. The inset scale bar represents 1 μm. *F*, detection of several mitochondrial stresses using mito-Pain T. HeLa cells expressing mito-Pain T were cultured with the indicated reagent for 24 h and then analyzed by flow cytometry. The value obtained by dividing the GFP/RFP of the compound treatment by GFP/RFP of the DMSO treatment is called PNIK1 stability. The data represent the mean ± SD (n = 3). ∗*p* < 0.05 and ∗∗*p* < 0.01. *G*, mito-Pain can distinguish cytoplasmic and mitochondrial accumulated PINK1-GFP. HeLa cells expressing mito-Pain T were cultured with MG-132 or DMSO for 6 h before fixation. The scale bar represents 5 μm. CCCP, carbonyl cyanide m-chlorophenyl hydrazone; DMSO, dimethyl sulfoxide; mito-Pain, mitochondrial PINK1 accumulation index; mito-Pain F, mitochondrial PINK1 accumulation index (containing full-length PINK1); mito-Pain T, truncated mito-Pain; MTS, mitochondrial targeting sequence; PINK1, PTEN-induced putative kinase 1; TMD, transmembrane domain.

Because PINK1 overexpression relieves mitochondrial stress and might be not convenient to use for stress detection (32, 33), we generated mito-Pain F with PINK1 kinase-dead (KD) cells. However, PINK1KD-GFP (19) showed very weak stability even after cell treatment with CCCP (data not shown). This finding is consistent with the previous finding that PINK1 is stabilized by self-oligomer formation *via* autophosphorylation (25). Accordingly, we replaced the kinase domain with the self-oligomer domain PB1 from PKCζ in mito-Pain (34, 35). Although PINK1ΔKD-PB1 showed improved GFP stability in cells treated CCCP, this mito-Pain variant was more resistant to retrotranslocation into the cytosol under basal conditions (data not shown). To facilitate degradation under basal conditions, we added the degron domain from Rpn4, which promotes proteasomal degradation (36, 37). The resultant plasmid (PINK1ΔKD-PB1-GFP-degron-T2A-RFP-Omp25TMD) is referred to as truncated mito-Pain (mito-Pain T) (Fig. 2*A*). The GFP-RFP ratio of mito-Pain T was 3.4-fold higher in CCCP-treated cells than in nontreated cells (Fig. 2, *B* and *C*), and the GFP signal of mito-Pain T upon CCCP treatment colocalized with RFP-Omp25 (Fig. 2*B*). These data suggest that mito-Pain T was more sensitive to mitochondrial stress than mito-Pain F. Notably, cells with high expression of mito-Pain T induced aggregation of GFP-positive mitochondria under mitochondrial stress conditions, possibly because of excessive multimerization capacity conferred through PB1 domains on mitochondria (Fig. 2*D*). To avoid mitochondrial aggregation, we cloned monoclonal HeLa cells stably expressing mito-Pain T at low levels, and these cells did not show aggregated mitochondria. Stabilized GFP and efficient cleavage at the T2A site in the cells stably expressing mito-Pain T were verified by immunoblotting (Fig. S1*A*). The GFP signal of mito-Pain T did not colocalize with the endoplasmic reticulum (ER), Golgi apparatus, or lysosomes and partially colocalized with peroxisomes, which is in a similar manner to other overexpressed membrane proteins mislocalized to incorrect organelles (Fig. 2*E*) (38).

To investigate the sensitivity of mito-Pain, we next quantified mitochondrial stress by known cell stress–inducing compounds using mito-Pain T. As expected, treatment with CCCP, oligomycin and antimycin (mitochondrial respiration inhibitors), and valinomycin (an ionophore) markedly elevated the GFP-RFP ratio to be more than 3.8-fold the level induced by dimethyl sulfoxide treatment (Fig. 2*F*).

Treatment with MG-132, a proteasome inhibitor, led to the greatest increase in the GFP-RFP ratio, which is 38.5-fold (Fig. 2*F*). MG-132 has been shown to induce caspase-mediated apoptosis with mitochondrial depolarization (39). However, considering proteasomal degradation of retrotranslocated PINK1 in the cytosol, the increase in the ratio induced by MG-132 was mainly due to a secondary effect. Fluorescence microscopy can be used to distinguish the accumulation of PINK1-GFP upon inhibition of proteasomal degradation or stabilization on mitochondria. Indeed, GFP accumulation both on mitochondria and in the cytosol after cell treatment with MG-132 was observed by fluorescence microscopy (Fig. 2*G*). Although PINK1 stability under thapsigargin treatment exhibited a marginal difference of ∼1.53 (Fig. 2*F*), fluorescence microscopic observation elucidated that PINK1-GFP localized to mitochondria (Fig. S1*B*). Taken together, the combination of flow cytometry and microscopy is a more reliable approach for analyzing mitochondrial stress using mito-Pain.

### Compound screening using mito-Pain T

**Figure 3:**
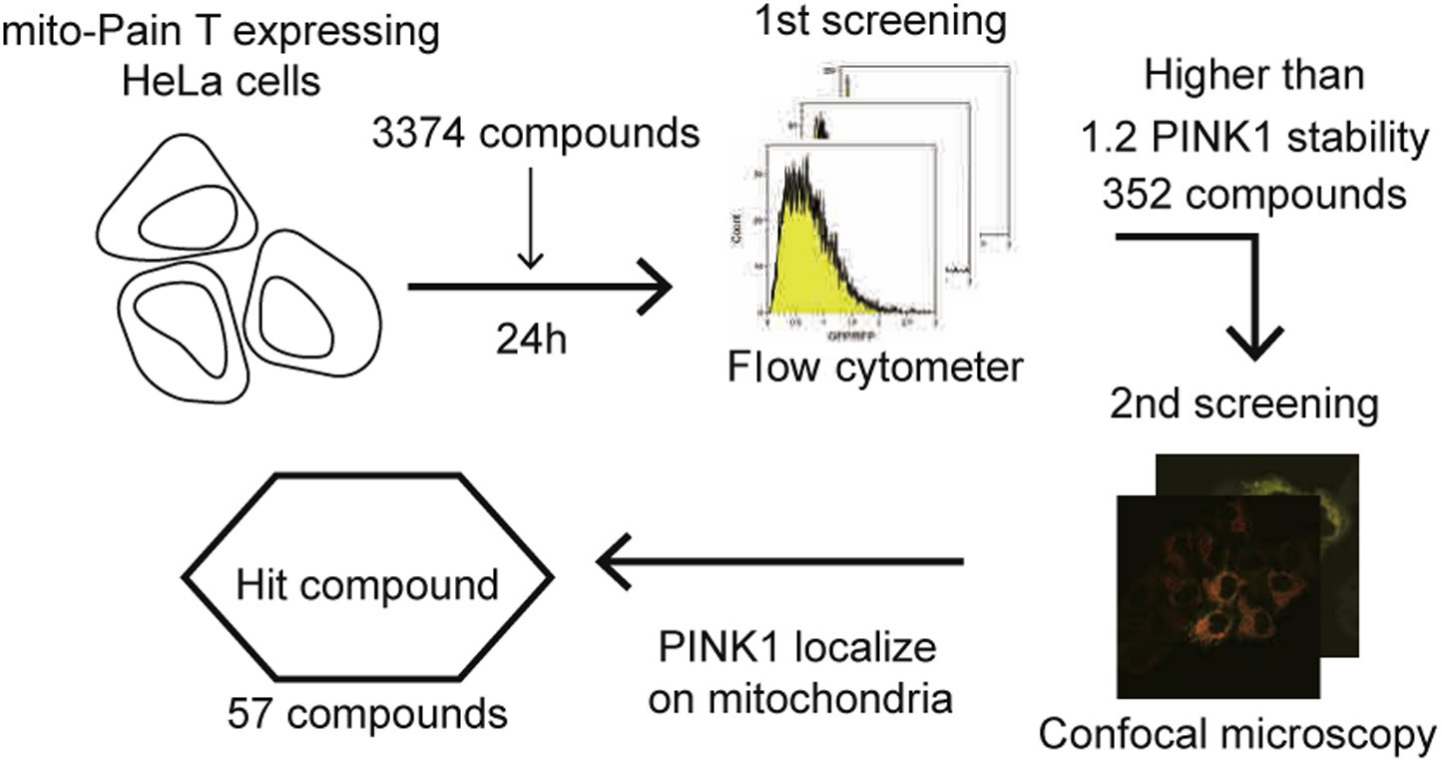
**Screening of mitochondrial stress–inducing compounds using mito-Pain T.** HeLa cells stably expressing mito-Pain T were treated with each compound obtained from a Validated Compound Library (3374 compounds) for 24 h before analysis. For the first screening, the GFP-RFP ratio was measured using flow cytometry. For the second screening, the localization of GFP was observed by fluorescence microscopy. Toxic compounds that induced cell death for 24 h were separately screened with a 5-h treatment, and these compounds are indicated by an *asterisk* in Table S1. mito-Pain, mitochondrial PINK1 accumulation index; mito-Pain T, truncated mito-Pain; PINK1, PTEN-induced putative kinase 1.

To identify novel mitochondrial stress inducers, we screened a Validated Compound Library (Drug Discovery Initiative) containing 3374 known compounds with mito-Pain T using flow cytometry and microscopy (Fig. 3). For the first screening, monoclonal HeLa cells expressing mito-Pain T were treated with each compound (10 μM) for 24 h, and then, the GFP-RFP ratio was measured by flow cytometry. From the first screening, 352 compounds showed elevated >1.2 PINK1 stability. For the second screening, we treated cells with each of the 352 compounds, and using fluorescence microscopy, we evaluated whether GFP localized to mitochondria or other compartments. Finally, we obtained 57 hit compounds considered to be mitochondrial stress inducers (Table S1).

To further characterize each phenotype, we selected one-half of the compounds on the basis of their functional properties. For example, among the hit compounds, compounds with higher PINK1 stability and a common function were selected, and in contrast, compounds that are difficult to purchase were not chosen. We purchased the selected 25 compounds separately from the compound library and confirmed reproducibility using mito-Pain T. The results showed that cell treatment with each of the selected compounds increased PINK1-GFP stability and induced PINK1-GFP localization on mitochondria, suggesting that these 25 compounds induce mitochondrial stress (Table 1). This new screening strategy is referred to as a mito-Pain assay.

**Table 1:** Summary of mitochondrial stress by identified compounds

Ranking	Compound	Function	Mitochondrial morphology	Dep.	Parkin	Reference
1	MG-115	20S and 26S proteasome inhibitor	Aggregation		Cytosol	(51)
2	Apicidin	HDAC inhibitor			Cytosol	(52, 53)
3	Hellebrin	Na^+^/K^+^-ATPase inhibitor	Aggregation		Cytosol	(54, 55)
4	Mocetinostat	HDAC1 inhibitor	Aggregation		Cytosol	(56)
5	β-Rubromycin	TERT	Fragmentation	✓^a^	Cytosol	-
6	Auranofin	TrxR inhibitor	Aggregation		Cytosol	(57, 58)
7	Azaguanine-8	Purine analog competing with guanine	Fragmentation		Cytosol	(59)
8	Rottlerin	PKC and CaM kinase III inhibitor	Aggregation	✓^b^	Mt	(60, 61)
9	Linifanib	Receptor tyrosine kinase inhibitor	Aggregation		Cytosol	-
10	Hexachlorophene	KCNQ1/KCNE1 potassium channel activator	Aggregation	✓^a^	Mt	(62)
11	Gossypol	Bcl-XL, Bcl-2, and Mcl-1 inhibitors	Swelling	✓^b^	Dots^d^	(63, 64)
12	Hyperforin	SIRT1 inhibitor	Aggregation	✓^b^	Mt	(65, 66)
13	Niclosamide	STAT3 inhibitor	Aggregation	✓^b^	Mt	(67, 68)
14	Chelerythrine	Bcl-XL-Bak binding inhibitor	Fragmentation	✓^b^	Cytosol	(69, 70)
15	JS-K	Generate NO	Elongation		Dots^c^	(71)
16	TOFA	Acetyl-CoA carboxylase inhibitor	Swelling		Cytosol	(72)
17	5-Azacytidine	DNA methyltransferase inhibitor			Cytosol	(73)
18	(R)-CR8	CDK1, 2, 5, 7, and 9 inhibitors	Fragmentation		Cytosol	(74)
19	Phorbol 12-myristate 13-acetate	PKC activator	Aggregation		Dots^c^	(75)
20	Deoxynivalenol	Bind to ribosome and inhibit protein synthesis			Cytosol	(76, 77)
21	Lexibulin	Tubulin polymerization inhibitor	Elongation		Cytosol	(78)
22	Thapsigargin	ER Ca^2+^-ATPases	Fragmentation		Dots^c^	(79, 80)
23	Vinorelbine	Tubulin polymerization inhibitor	Elongation		Dots^d^	(81, 82)
24	Podophyllotoxin	Tubulin polymerization inhibitor	Elongation		Cytosol	(83)
25	Spiperone	Ca^2+^-activated Cl^−^channel activator			Dots^d^	(84)

Abbreviations: Dep., mitochondrial depolarization; Mt, mitochondria; Parkin, Parkin localization; TrxR, thioredoxin reductase.

a Depolarized in the MTR or JC-1 assay.

b Depolarized in both the MTR and JC-1 assay.

c Partial mitochondrial localization of Parkin puncta.

d Cytosolic localization of Parkin puncta.

### Twenty-one of 25 hit compounds affected mitochondrial morphology

**Figure 4:**
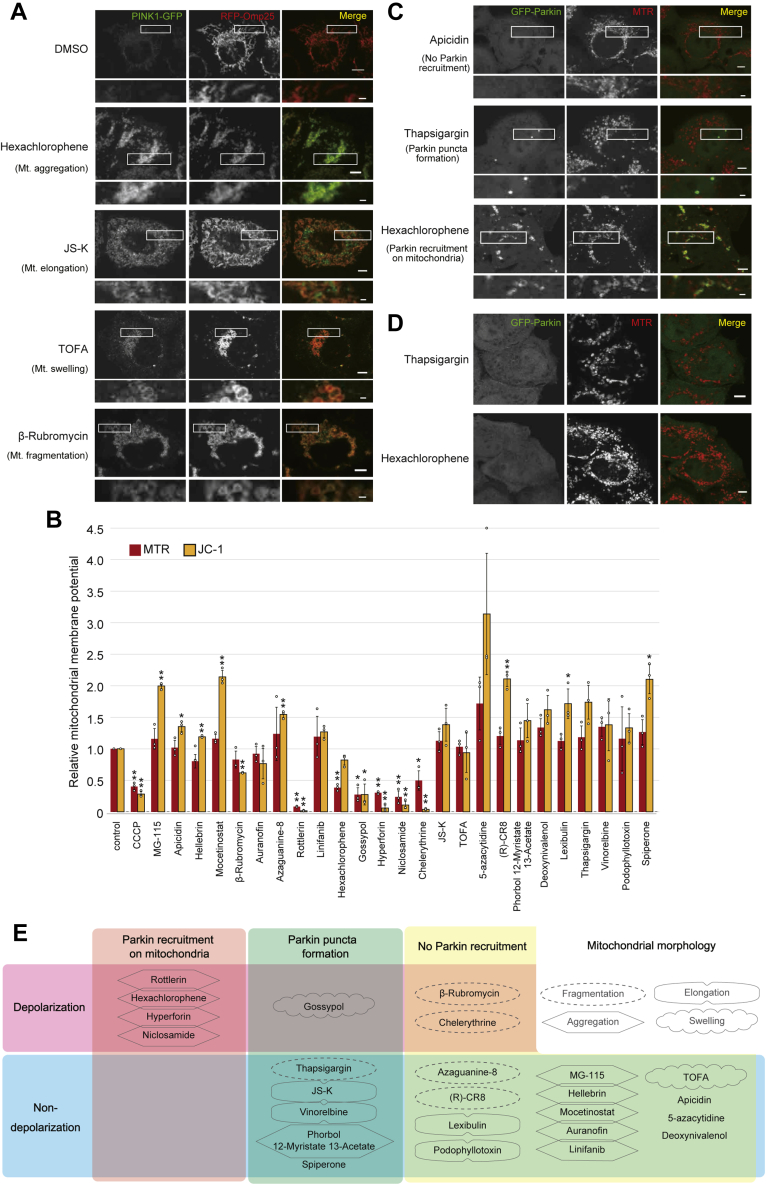
**Characterization of mitochondrial stresses by hit compounds.***A*, representative mitochondrial morphological changes by hit compounds. HeLa cells stably expressing mito-Pain T were cultured with the indicated compounds for 24 h before fixation and observed by fluorescence microscopy. The scale bar represents 5 μm. The inset scale bar represents 1 μm. *B*, measurement of mitochondrial depolarization using MTR or JC-1. For MTR, HeLa cells expressing PINK1-GFP were cultured with the indicated compounds for 24 h or 6 h and then treated with MTR for 15 min before analysis. For JC-1, HeLa cells were cultured with the indicated compounds for 24 h or 6 h and then treated with JC-1 for 30 min before analysis. Note: some compounds (MG-115, apicidin, hellebrin, mocetinostat, β-rubromycin, auranofin, azaguanine-8, linifanib, hexachlorophene, chelerythrine, JS-K, (R)-CR8, phorbol 12-myristate 13-acetate, deoxynivalenol, lexibulin, thapsigargin, vinorelbine, podophyllotoxin, and spiperone) that are toxic for longer treatment time were incubated with cells for 6 h. The MTR or JC-1 fluorescence intensities were measured by flow cytometry. The data represent the mean ± SD (n = 3). ∗*p* < 0.05 and ∗∗*p* < 0.01. *C*, some compounds changed Parkin distribution. HeLa cells expressing GFP-Parkin were pretreated with MTR for 15 min and then cultured with the indicated compounds for 6 h before fixation and observed by fluorescence microscopy. The scale bar represents 5 μm. The inset scale bar represents 1 μm. *D*, PINK1 is essential for Parkin recruitment by hit compounds. PINK1 KO HeLa cells expressing GFP-Parkin were cultured with the indicated compounds for 6 h before fixation and observed by fluorescence microscopy. The scale bar represents 5 μm. *E*, groups of hit compounds as categorized by depolarization and Parkin recruitment. CCCP, carbonyl cyanide m-chlorophenyl hydrazone; DMSO, dimethyl sulfoxide; mito-Pain, mitochondrial PINK1 accumulation index; mito-Pain T, truncated mito-Pain; MTR, MitoTracker Red; PINK1, PTEN-induced putative kinase 1.

As stressed mitochondria often affect mitochondrial fusion, fission, and membrane structure (40), mitochondrial morphological changes are among the indicators of mitochondrial stress. RFP-Omp25 in mito-Pain can be simultaneously applied for analysis of mitochondrial morphology. We observed cells expressing mito-Pain T treated with the hit compounds by fluorescence microscopy. A variety of abnormal mitochondrial morphologies, including fragmented, elongated, aggregated, and swelled mitochondria, were confirmed in 21 of 25 hit compounds (Table 1) (Fig. 4*A*). Aggregated mitochondria were observed in cells treated with MG-115, hellebrin, mocetinostat, auranofin, rottlerin, linifanib, hexachlorophene, hyperforin, niclosamide, or phorbol 12-myristate 13-acetate (PMA); fragmented mitochondria were observed in cells treated with β-rubromycin, azaguanine-8, chelerythrine, (R)-CR8, or thapsigargin; elongated mitochondria were observed in cells treated with JS-K, lexibulin, vinorelbine, or podophyllotoxin; swelled mitochondria were observed in cells treated with gossypol or TOFA. These data indicate that PINK1-GFP stability in mito-Pain is a response to various mitochondrial stresses. Unexpectedly, PINK1-GFP was not always localized on mitochondria. For example, PINK1-GFP stabilized by JS-K, lexibulin, and vinorelbine treatment was observed on both mitochondria and nonmitochondrial compartments (Figs. 4*A* and S2*A*). As lexibulin and vinorelbine are tubulin polymerization inhibitors, we also observed mito-Pain F after using the general tubulin polymerization inhibitors nocodazole and colchicine, which also led to nonmitochondrial localization of PINK1 (Fig. S2*B*). In addition, we examined the localization of PINK1 to microtubule-organizing organelles, including the ER, Golgi apparatus, and lysosomes (Fig. S2*C*). Under vinorelbine treatment, nonmitochondrial PINK1 did not localize to the ER or Golgi apparatus, and only a few PINK1 were partially localized to lysosomes, suggesting that PINK1 might function on mitochondria and nonmicrotubule-organizing compartments under tubulin polymerization inhibitor treatment. Spiperone also induced unique localization of PINK1, which formed punctate structures localized to mitochondria (Fig. S2*D*).

### Eighteen of 25 hit compounds induced mitochondrial stress independent of depolarization

Because depolarization leads to recruitment of PINK1 to mitochondria, we examined whether the hit compounds are associated with depolarization. We measured depolarization with MitoTracker Red (MTR), which is selectively retained in mitochondria with an intact membrane potential, and JC-1, which stains polarized mitochondria in red and depolarized mitochondria in green. Interestingly, only seven of the 25 compounds, including *β*-rubromycin, rottlerin, hexachlorophene, gossypol, hyperforin, niclosamide, and chelerythrine, considerably reduced the membrane potential (Table 1 and Fig. 4*B*). These results strongly indicate that three-quarters of the hit compounds induced mitochondrial stress *via* depolarization-independent mechanisms.

### PINK1 functioned in mitochondria independent of Parkin under some mitochondrial stresses

As we mentioned above, Parkin recruitment to mitochondria is essential for PINK1-mediated mitophagy. Therefore, we examined the recruitment of Parkin by treatment with hit compounds using HeLa cells stably expressing GFP-Parkin. As 24 h of treatment with the compounds might diminish Parkin-recruited mitochondria by mitophagic degradation, we first treated the cells for 6 h to observe GFP-Parkin recruitment. We next tried to observe GFP-Parkin for 24-h treatment with the compounds, which did not induce GFP-Parkin recruitment for 6-h treatment. Rottlerin, hexachlorophene, hyperforin, and niclosamide induced recruitment of Parkin to mitochondria (Table 1). Unexpectedly, some compounds led to GFP-Parkin puncta formation (Figs. 4*C* and S3*A*). JS-K, PMA, and thapsigargin induced punctate structures of GFP-Parkin in both the mitochondria and cytosol, whereas gossypol, vinorelbine, and spiperone led to puncta formation only in the cytosol. By treating PINK1-KO HeLa cells with the compounds, we confirmed that PINK1 was necessary for all Parkin recruitment (localization on mitochondria and puncta formation) (Figs. 4*D* and S3*B*). These data imply that PINK1 responds to mitochondrial stresses *via* not only Parkin-dependent mechanisms but also Parkin-independent mechanisms.

## Discussion

In this study, we developed a novel sensor, mito-Pain, that allowed us to quantitatively measure various mitochondrial stresses. We applied the sensor to compound screening and identified several types of mitochondrial stress inducers. Many dyes are available for mitochondrial staining, most of which are dependent on mitochondrial membrane potential (12, 13). Therefore, these dyes were used to detect mitochondrial stress. However, it has been shown that mitochondrial stress is not always associated with mitochondrial depolarization (10). Although another classical indicator of mitochondrial stress is mitochondrial morphology, which dynamically changes to fragmentation, elongation, or swelling by stress (41, 42), it is difficult to measure morphological changes quantitatively.

We generated mito-Pain based on PINK1-GFP, which accumulates on the OMM under impairment of mitochondrial translocons. As mito-Pain coexpresses RFP-Omp25 as an internal control, it can be used to quantify the stress level *via* ratiometric analysis of GFP and RFP. Mito-Pain T was more sensitive to mitochondrial stress than mito-Pain F, but mito-Pain T tended to cause mitochondrial aggregation. Therefore, mito-Pain F is recommended for use for microscopic observations, and mito-Pain T is recommended for use in flow cytometric analysis. Through our compound screening, we identified various types of mitochondrial stress inducers. Interestingly, these compounds showed different mitochondrial stress responses, including mitochondrial depolarization, morphological changes, and Parkin recruitment (Table 1), indicating that mito-Pain is a more versatile tool for detecting various mitochondrial stresses than mitochondrial membrane potential–dependent dyes. Another advantage of mito-Pain over mitochondrial membrane potential–dependent dyes is that GFP specifically labels stressed mitochondria but not healthy mitochondria (Fig. 1*E*). This distinction is useful for observing partially stressed mitochondria in a cell. Indeed, mito-Pain detected partial PINK1-GFP–positive mitochondria induced by spiperone (Fig. S2*D*). In addition, although flow cytometric analysis did not show a significant difference with thapsigargin treatment (Fig. 2*F*), microscopic analysis revealed that PINK1-GFP localized to mitochondria under thapsigargin treatment (Fig. S1*B*). This is consistent with the result that thapsigargin impacted Parkin localization in a manner dependent on PINK1 (Fig. 4*C*). Therefore, combination analysis with flow cytometry and microscopy using mito-Pain was able to detect a broad range of mitochondrial stresses. Although MG-132, a proteasome inhibitor, dramatically increased the GFP-RFP ratio, this was mainly a secondary effect in which PINK1-GFP accumulated both in the cytosol and in mitochondria (Fig. 2*G*). As a result of compound screening using mito-Pain T, we focused on and confirmed the reproducible effects of 25 compounds as mitochondrial stress inducers. Considering that 23 of the 25 compounds have been reported to affect mitochondrial function and that 21 of the 25 compounds affected mitochondrial morphology (Table 1), mito-Pain assay is an efficient mitochondrial stress detection strategy.

Among the 25 compounds, β-rubromycin, azaguanine-8, linifanib, and spiperone have not been characterized with molecular mechanisms that cause stress on mitochondria. β-Rubromycin is an inhibitor of telomerase reverse transcriptase (TERT). TERT not only functions in telomere elongation but also inhibits caspase-mediated apoptosis and generates ROS in mitochondria (43), suggesting that TERT inhibition induces mitochondrial stress independently of telomeres. Azaguanine-8 is a purine analog showing antineoplastic activity by competing with guanine. We speculate that azaguanine-8 is incorporated into mtDNA during replication, leading to mtDNA damage that might induce mitochondrial stress. Linifanib is a receptor tyrosine kinase (RTK) inhibitor. Other RTK inhibitors, namely, sorafenib and imatinib, inhibit not only RTK but also respiration chains in mitochondria. Furthermore, lapatinib inhibits glycolysis, and sunitinib reduces mitochondrial membrane potential (44, 45), suggesting that RTK inhibitor structures endow them with affinity for mitochondria. Therefore, linifanib might inhibit mitochondrial function in a manner similar to other RTK inhibitors. On the other hand, linifanib increases the mRNA levels of mitochondrial proteins and respiration (46), but it did not cause mitochondrial depolarization (Table 1) or parkin recruitment in our study (Fig. 4*E*), suggesting that linifanib recruited PINK1 to mitochondria for mitochondrial repair but not mitophagy. Spiperone is an antagonist of a serotonin receptor, a dopamine receptor, and a Ca^2+^-activated Cl^−^ channel (TMEM16A/Ano1) activator (47). Because the expression of these receptors and channels is restricted to certain tissues, the HeLa cells we used in this study might not express these proteins (48). Mitochondrial stress induced by spiperone is mediated *via* an unknown target.

Inhibition of tubulin polymerization induced mitochondrial elongation and nonmitochondrial localization of PINK1 (Fig. S2, *A* and *B*), implying that elongated mitochondria might affect nonmitochondrial compartments that recruit PINK1 *via* an unknown mechanism. Not all elongation inducers induced nonmitochondrial localization of PINK1 (*e.g.*, podophyllotoxin), but JS-K, which is not a tubulin inhibitor, also induced nonmitochondrial localization of PINK1 with mitochondrial elongation (Fig. 4, *A* and *E*), suggesting that mitochondrial elongation might be associated with recruitment of PINK1 to nonmitochondrial compartments. Further studies will elucidate crosstalk between elongated mitochondria and PINK1-positive nonmitochondrial compartments.

Parkin is an essential factor for PINK1-mediated mitophagy. We investigated the localization of Parkin under hit compound treatment (Fig. 4*C* and Table 1). Four compounds, rottlerin, hexachlorophene, hyperforin, or niclosamide, showed apparent parkin localization on mitochondria and induced mitochondrial depolarization. These findings are consistent with the fact that depolarization induces mitophagy (49). Unexpectedly, six compounds, gossypol, JS-K, PMA, thapsigargin, vinorelbine, and spiperone, induced Parkin puncta formation (Figs. 4*C* and S3*A* and Table 1). As Parkin puncta formation was dependent on PINK1 (Figs. 4*D* and S3*B*), the Parkin puncta formation might be mediated by PINK1 activity and represents a kind of stress response. Although there were no common mitochondrial morphological changes induced by these compounds, Parkin puncta after PMA, thapsigargin, or JS-K treatment colocalized in some mitochondria, indicating that the Parkin puncta induced partial mitophagy. The other three compounds (gossypol, vinorelbine, and spiperone) induced Parkin puncta formation in the cytosolic region. Because Parkin localization to ectopic sites was dependent on PINK1 (Figs. 4*D* and S3*B*), Parkin might relocate from PINK1-positive mitochondria to these sites. One possibility is that mitochondria stressed by these compounds trigger damage expansion into other regions, leading to Parkin relocation to ectopic sites. In contrast, the other 15 compounds did not affect Parkin localization despite PINK1 accumulation on mitochondria. PINK1 might function in the repair of damaged mitochondria but not in the degradation of stressed mitochondria by mitophagy. Indeed, PINK1 has some functions independent of Parkin (26, 50). In that case, Parkin might not be recruited to PINK1-positive mitochondria to avoid mitophagy.

These results imply that PINK1 has two distinct roles as a mitochondrial quality control system. In one role, PINK1 removes damaged mitochondria *via* Parkin-mediated mitophagy when mitochondria are heavily damaged (*e.g.*, depolarization). In the other role, PINK1 rescues damaged mitochondria *via* unidentified factor(s) when mitochondria are partially damaged. Collectively, mito-Pain used in our study provides a versatile method to quantitatively measure various mitochondrial stresses compared with currently used mitochondrial dyes. Furthermore, the mitochondrial stress inducers identified in this study will provide new insight into the fundamental understanding of mitochondrial quality control systems.

## Experimental procedures

### Cell culture

HeLa, COS, and HEK293FT (HEK293) cells were cultured in Dulbecco’s modified Eagle’s medium (Nacalai Tesque) supplemented with 10% fetal bovine serum (MP Bio) and 50 mg/ml penicillin and streptomycin (regular medium) in a humidified atmosphere with 5% CO_2_ at 37 °C. For compound treatment, cells were incubated for the indicated times and concentrations, as listed in Table S2. For experiments involving mitochondrial staining by MitoTracker Red CMXROS (product No. M7512), purchased from Thermo Fisher Scientific, HeLa cells were incubated with 0.125 μM MTR for 15 min or 2.0 μM JC-1 for 30 min for use in flow cytometry and 0.2 μM MTR for 15 min for use in fluorescence microscopy.

### Plasmids

To generate the constructs pLenti-puro PINK1-sfGFP-T2A-mScarlet i-Omp25, pLenti-puro PINK1ΔKD-PB1-sfGFP-Rpn4-T2A-mScarlet i-Omp25, pLenti-puro PINK1-sfGFP, and pMRX GFP-Parkin, PINK1 (amino acid residues 1–581 for mito-Pain F and amino acid residues 1–152 for mito-Pain T) was amplified from pLenti6-DEST PINK1-V5 WT (Addgene; plasmid #13320). The PB1 domain (PKCζ) (amino acid residues 15–98) was amplified from HeLa total cDNA, Omp25 (amino acid residues 107–146) was amplified from HEK293 total cDNA, Parkin (human) was amplified from YFP-Parkin (Addgene; plasmid #23955), and Rpn4 (amino acid residues 178–327) was amplified from *Saccharomyces cerevisiae* (BY4741) genomic DNA. The PCR products were inserted into a pCW 57.1 (Addgene; plasmid #41393) or pLenti CMV GFP Puro (Addgene; plasmid #17448) plasmid together with superfolder GFP, the T2A peptide sequence, and mScarlet-I *via* Gibson assembly.

### Generation of KO cells using CRISPR

Human PINK1 sgRNA 5′- GTCGCACCGCCATGGTGGCG -3′ was cloned into a lentiCRISPRv2 hygro plasmid (Addgene; plasmid #98291). HeLa cells were infected with lentivirus harboring sgRNA and Cas9. Hygromycin was added to the cells after 24 h of infection. After culture for >7 days, the cells were used as a KO cell line.

### Antibodies

A rabbit polyclonal antibody against Trapα was a gift from R.S. Hegde (MRC LMB). A rabbit polyclonal antibody against Lamp1 was a gift from Y. Tanaka (Kyushu University). A rabbit polyclonal antibody against Tom20 (code No. sc-11415) was purchased from Santa Cruz Biotechnology. Rabbit polyclonal antibody against GM130 (code No. PM061) was purchased from MBL. A rabbit polyclonal antibody against Pex19 (catalog No. 10594-1-AP) was purchased from Proteintech. Rabbit polyclonal anti-GFP and anti-RFP were generated using recombinant proteins. Mouse monoclonal anti-β-actin (clone 2F3, cat. No. 281-98721) antibody was purchased from Wako Chemicals. Rabbit polyclonal anti-PINK1 (cat. No. BC100-494SS) antibody was purchased from Novus Biologicals.

### Generation of stable cell lines by lentiviral and retroviral infection

For preparation of lentiviruses and retroviruses, HEK293FT cells were transiently cotransfected with lentiviral (pLenti-puro or pLenti-hygro with pCMV-VSVG (Addgene; plasmid #8454) and psPAX2 (Addgene; plasmid #12260)) or retroviral (pMRX GFP-Parkin with pCMV-VSVG (Addgene; plasmid #8454) and Gag) vectors using PEI MAX reagent (Polysciences). After culturing for 72 h, the growth medium containing the virus was centrifuged, and the resultant supernatant was collected. HeLa cells were incubated with this collected virus-containing medium with 10 mg/ml polybrene for 24 h and then selected with 1 μg/ml puromycin (InvivoGen) or 100 μg/ml hygromycin.

### Immunocytochemistry and fluorescence microscopy

Cells grown on coverslips were washed with PBS, fixed with 3.7% formaldehyde in PBS for 15 min, and observed under a confocal laser microscope (FV1000 IX81; Olympus) using a 60× and 100× oil-immersion objective lens with a numerical aperture of 1.40. For immunostaining, fixed cells were permeabilized with 0.1% Triton X-100 or 50 μg/ml digitonin in PBS for 5 min, blocked with 10% newborn bovine serum in PBS for 30 min, and incubated with primary antibodies for 1 h. After washing, the cells were incubated with Alexa Fluor 647–conjugated goat anti-rabbit IgG secondary antibodies (Thermo Fisher Scientific) for 1 h.

### Flow cytometry

Trypsinized cells were passed through a 70-μm cell strainer, resuspended in 5% newborn bovine serum and 1 μg/ml 4′,6-diamidino-2-phenylindole (DAPI) in PBS, and then analyzed by a CytoFLEX S flow cytometer equipped with NUV 375-nm (DAPI), 488-nm (GFP), and 561-nm (RFP) lasers (Beckman Coulter). DAPI-positive cells were removed as dead cells. In each sample, 10,000 cells were acquired. The data were processed with Kaluza software (Beckman Coulter).

### Compound screening

Validated Compound Library of 3374 compounds provided by the Drug Discovery Initiative (The University of Tokyo) was used for screening. HeLa cells stably expressing mito-Pain T were cultured in a regular medium with 10 μM compound for 24 h. For the first screening, the GFP-RFP fluorescence ratio was measured using a flow cytometer, and then, the number of compounds was reduced to 352 candidates with a GFP-RFP ratio greater than 1.20. For the second screening, the cellular localization of PINK1-GFP to the mitochondrial structures after compound treatment was examined using confocal microscopy. Compounds representing PINK1-GFP–positive mitochondria were identified as hit compounds.

### Immunoblotting

Cells were washed with cold PBS and lysed in the lysis buffer (1% Triton X-100; 50 mM Tris/HCl, pH 7.5; 1 mM EDTA; and 150 mM NaCl) supplemented with protease-inhibitor cocktail (EDTA-free) (Nacalai Tesque) and 1 mM PMSF for 15 min at 4 °C. The lysates were clarified by centrifugation at 20,630*g* for 5 min, and the SDS sample buffer was added. The samples were boiled at 95 °C for 5 min before being subjected to SDS/PAGE. Twenty micrograms of protein per lane was separated by SDS-PAGE and then transferred to a polyvinylidene difluoride membrane (Millipore). Immunoblot analysis was performed with the indicated antibodies, and the immunoreactive proteins were visualized using ImmunoStar Zeta (Wako Chemicals).

## Data availability

All experimental data is included in the article.

## Supporting information

This article contains supporting information.

## Conflict of interest

The authors declare that they have no conflicts of interest with the contents of this article.

## References

[1] Saraste M. (1999). Oxidative phosphorylation at the fin de siècle. Science.

[2] Li X., Fang P., Mai J., Choi E.T., Wang H., Yang X.F. (2013). Targeting mitochondrial reactive oxygen species as novel therapy for inflammatory diseases and cancers. J. Hematol. Oncol..

[3] Zorov D.B., Juhaszova M., Sollott S.J. (2014). Mitochondrial reactive oxygen species (ROS) and ROS-induced ROS release. Physiol. Rev..

[4] Chen J.J., Yu B.P. (1994). Alterations in mitochondrial membrane fluidity by lipid peroxidation products. Free Radic. Biol. Med..

[5] Shokolenko I., Venediktova N., Bochkareva A., Wilson G.L., Alexeyev M.F. (2009). Oxidative stress induces degradation of mitochondrial DNA. Nucleic Acids Res..

[6] Bota D.A., Davies K.J. (2002). Lon protease preferentially degrades oxidized mitochondrial aconitase by an ATP-stimulated mechanism. Nat. Cell Biol..

[7] Nargund A.M., Pellegrino M.W., Fiorese C.J., Baker B.M., Haynes C.M. (2012). Mitochondrial import efficiency of ATFS-1 regulates mitochondrial UPR activation. Science.

[8] Weidberg H., Amon A. (2018). MitoCPR-A surveillance pathway that protects mitochondria in response to protein import stress. Science.

[9] Pickles S., Vigié P., Youle R.J. (2018). Mitophagy and quality control mechanisms in mitochondrial maintenance. Curr. Biol..

[10] Jin S.M., Youle R.J. (2013). The accumulation of misfolded proteins in the mitochondrial matrix is sensed by PINK1 to induce PARK2/Parkin-mediated mitophagy of polarized mitochondria. Autophagy.

[11] Palikaras K., Daskalaki I., Markaki M., Tavernarakis N. (2017). Mitophagy and age-related pathologies: Development of new therapeutics by targeting mitochondrial turnover. Pharmacol. Ther..

[12] Scaduto R.C., Grotyohann L.W. (1999). Measurement of mitochondrial membrane potential using fluorescent rhodamine derivatives. Biophys. J..

[13] Poot M., Zhang Y.Z., Krämer J.A., Wells K.S., Jones L.J., Hanzel D.K., Lugade A.G., Singer V.L., Haugland R.P. (1996). Analysis of mitochondrial morphology and function with novel fixable fluorescent stains. J. Histochem. Cytochem..

[14] Han J., Goldstein L.A., Gastman B.R., Rabinowich H. (2006). Interrelated roles for Mcl-1 and BIM in regulation of TRAIL-mediated mitochondrial apoptosis. J. Biol. Chem..

[15] Rehling P., Wiedemann N., Pfanner N., Truscott K.N. (2001). The mitochondrial import machinery for preproteins. Crit. Rev. Biochem. Mol. Biol..

[16] Kitada T., Asakawa S., Hattori N., Matsumine H., Yamamura Y., Minoshima S., Yokochi M., Mizuno Y., Shimizu N. (1998). Mutations in the parkin gene cause autosomal recessive juvenile parkinsonism. Nature.

[17] Valente E.M., Abou-Sleiman P.M., Caputo V., Muqit M.M., Harvey K., Gispert S., Ali Z., Del Turco D., Bentivoglio A.R., Healy D.G., Albanese A., Nussbaum R., González-Maldonado R., Deller T., Salvi S. (2004). Hereditary early-onset Parkinson's disease caused by mutations in PINK1. Science.

[18] Koyano F., Okatsu K., Kosako H., Tamura Y., Go E., Kimura M., Kimura Y., Tsuchiya H., Yoshihara H., Hirokawa T., Endo T., Fon E.A., Trempe J.F., Saeki Y., Tanaka K. (2014). Ubiquitin is phosphorylated by PINK1 to activate parkin. Nature.

[19] Okatsu K., Oka T., Iguchi M., Imamura K., Kosako H., Tani N., Kimura M., Go E., Koyano F., Funayama M., Shiba-Fukushima K., Sato S., Shimizu H., Fukunaga Y., Taniguchi H. (2012). PINK1 autophosphorylation upon membrane potential dissipation is essential for Parkin recruitment to damaged mitochondria. Nat. Commun..

[20] Shiba-Fukushima K., Imai Y., Yoshida S., Ishihama Y., Kanao T., Sato S., Hattori N. (2012). PINK1-mediated phosphorylation of the Parkin ubiquitin-like domain primes mitochondrial translocation of Parkin and regulates mitophagy. Sci. Rep..

[21] Jin S.M., Lazarou M., Wang C., Kane L.A., Narendra D.P., Youle R.J. (2010). Mitochondrial membrane potential regulates PINK1 import and proteolytic destabilization by PARL. J. Cell Biol..

[22] Yamano K., Youle R.J. (2013). PINK1 is degraded through the N-end rule pathway. Autophagy.

[23] Meissner C., Lorenz H., Weihofen A., Selkoe D.J., Lemberg M.K. (2011). The mitochondrial intramembrane protease PARL cleaves human Pink1 to regulate Pink1 trafficking. J. Neurochem..

[24] Narendra D.P., Jin S.M., Tanaka A., Suen D.F., Gautier C.A., Shen J., Cookson M.R., Youle R.J. (2010). PINK1 is selectively stabilized on impaired mitochondria to activate Parkin. PLoS Biol..

[25] Okatsu K., Uno M., Koyano F., Go E., Kimura M., Oka T., Tanaka K., Matsuda N. (2013). A dimeric PINK1-containing complex on depolarized mitochondria stimulates Parkin recruitment. J. Biol. Chem..

[26] Huang E., Qu D., Huang T., Rizzi N., Boonying W., Krolak D., Ciana P., Woulfe J., Klein C., Slack R.S., Figeys D., Park D.S. (2017). PINK1-mediated phosphorylation of LETM1 regulates mitochondrial calcium transport and protects neurons against mitochondrial stress. Nat. Commun..

[27] Kühn K., Zhu X.R., Lübbert H., Stichel C.C. (2004). Parkin expression in the developing mouse. Brain Res. Dev. Brain Res..

[28] Unoki M., Nakamura Y. (2001). Growth-suppressive effects of BPOZ and EGR2, two genes involved in the PTEN signaling pathway. Oncogene.

[29] Kim J.H., Lee S.R., Li L.H., Park H.J., Park J.H., Lee K.Y., Kim M.K., Shin B.A., Choi S.Y. (2011). High cleavage efficiency of a 2A peptide derived from porcine teschovirus-1 in human cell lines, zebrafish and mice. PLoS One.

[30] Donnelly M.L., Luke G., Mehrotra A., Li X., Hughes L.E., Gani D., Ryan M.D. (2001). Analysis of the aphthovirus 2A/2B polyprotein 'cleavage' mechanism indicates not a proteolytic reaction, but a novel translational effect: A putative ribosomal 'skip'. J. Gen. Virol..

[31] Nemoto Y., De Camilli P. (1999). Recruitment of an alternatively spliced form of synaptojanin 2 to mitochondria by the interaction with the PDZ domain of a mitochondrial outer membrane protein. EMBO J..

[32] Wen Y., Gu Y., Tang X., Hu Z. (2020). PINK1 overexpression protects against cerebral ischemia through Parkin regulation. Environ. Toxicol..

[33] Sánchez-Mora R.M., Arboleda H., Arboleda G. (2012). PINK1 overexpression protects against C2-ceramide-induced CAD cell death through the PI3K/AKT pathway. J. Mol. Neurosci..

[34] Wilson M.I., Gill D.J., Perisic O., Quinn M.T., Williams R.L. (2003). PB1 domain-mediated heterodimerization in NADPH oxidase and signaling complexes of atypical protein kinase C with Par6 and p62. Mol. Cell.

[35] Sumimoto H., Kamakura S., Ito T. (2007). Structure and function of the PB1 domain, a protein interaction module conserved in animals, fungi, amoebas, and plants. Sci. STKE.

[36] Erales J., Coffino P. (2014). Ubiquitin-independent proteasomal degradation. Biochim. Biophys. Acta.

[37] Morozov A.V., Spasskaya D.S., Karpov D.S., Karpov V.L. (2014). The central domain of yeast transcription factor Rpn4 facilitates degradation of reporter protein in human cells. FEBS Lett..

[38] Setoguchi K., Otera H., Mihara K. (2006). Cytosolic factor- and TOM-independent import of C-tail-anchored mitochondrial outer membrane proteins. EMBO J..

[39] Guo N., Peng Z. (2013). MG132, a proteasome inhibitor, induces apoptosis in tumor cells. Asia Pac. J. Clin. Oncol..

[40] Yu T., Robotham J.L., Yoon Y. (2006). Increased production of reactive oxygen species in hyperglycemic conditions requires dynamic change of mitochondrial morphology. Proc. Natl. Acad. Sci. U. S. A..

[41] Lakroun Z., Kebieche M., Lahouel A., Zama D., Desor F., Soulimani R. (2015). Oxidative stress and brain mitochondria swelling induced by endosulfan and protective role of quercetin in rat. Environ. Sci. Pollut. Res. Int..

[42] Wu S., Zhou F., Zhang Z., Xing D. (2011). Mitochondrial oxidative stress causes mitochondrial fragmentation via differential modulation of mitochondrial fission-fusion proteins. FEBS J..

[43] Low K.C., Tergaonkar V. (2013). Telomerase: Central regulator of all of the hallmarks of cancer. Trends Biochem. Sci..

[44] Paech F., Bouitbir J., Krähenbühl S. (2017). Hepatocellular toxicity associated with tyrosine kinase inhibitors: Mitochondrial damage and inhibition of glycolysis. Front. Pharmacol..

[45] Will Y., Dykens J.A., Nadanaciva S., Hirakawa B., Jamieson J., Marroquin L.D., Hynes J., Patyna S., Jessen B.A. (2008). Effect of the multitargeted tyrosine kinase inhibitors imatinib, dasatinib, sunitinib, and sorafenib on mitochondrial function in isolated rat heart mitochondria and H9c2 cells. Toxicol. Sci..

[46] Zhao S., Chu Y., Zhang Y., Zhou Y., Jiang Z., Wang Z., Mao L., Li K., Sun W., Li P., Jia S., Wang C., Xu A., Loomes K., Tang S. (2019). Linifanib exerts dual anti-obesity effect by regulating adipocyte browning and formation. Life Sci..

[47] Liang L., MacDonald K., Schwiebert E.M., Zeitlin P.L., Guggino W.B. (2009). Spiperone, identified through compound screening, activates calcium-dependent chloride secretion in the airway. Am. J. Physiol. Cell Physiol..

[48] Guo S., Chen Y., Pang C., Wang X., Shi S., Zhang H., An H., Zhan Y. (2019). Matrine is a novel inhibitor of the TMEM16A chloride channel with antilung adenocarcinoma effects. J. Cell Physiol..

[49] Narendra D., Tanaka A., Suen D.F., Youle R.J. (2008). Parkin is recruited selectively to impaired mitochondria and promotes their autophagy. J. Cell Biol..

[50] Tain L.S., Chowdhury R.B., Tao R.N., Plun-Favreau H., Moisoi N., Martins L.M., Downward J., Whitworth A.J., Tapon N. (2009). Drosophila HtrA2 is dispensable for apoptosis but acts downstream of PINK1 independently from Parkin. Cell Death Differ..

[51] Nam Y.J., Lee D.H., Lee M.S., Lee C.S. (2015). K(ATP) channel block prevents proteasome inhibitor-induced apoptosis in differentiated PC12 cells. Eur. J. Pharmacol..

[52] Cheong J.W., Chong S.Y., Kim J.Y., Eom J.I., Jeung H.K., Maeng H.Y., Lee S.T., Min Y.H. (2003). Induction of apoptosis by apicidin, a histone deacetylase inhibitor, via the activation of mitochondria-dependent caspase cascades in human Bcr-Abl-positive leukemia cells. Clin. Cancer Res..

[53] Kwon S.H., Ahn S.H., Kim Y.K., Bae G.U., Yoon J.W., Hong S., Lee H.Y., Lee Y.W., Lee H.W., Han J.W. (2002). Apicidin, a histone deacetylase inhibitor, induces apoptosis and Fas/Fas ligand expression in human acute promyelocytic leukemia cells. J. Biol. Chem..

[54] Daniel D., Süsal C., Kopp B., Opelz G., Terness P. (2003). Apoptosis-mediated selective killing of malignant cells by cardiac steroids: Maintenance of cytotoxicity and loss of cardiac activity of chemically modified derivatives. Int. Immunopharmacol..

[55] Deng L.J., Hu L.P., Peng Q.L., Yang X.L., Bai L.L., Yiu A., Li Y., Tian H.Y., Ye W.C., Zhang D.M. (2014). Hellebrigenin induces cell cycle arrest and apoptosis in human hepatocellular carcinoma HepG2 cells through inhibition of Akt. Chem. Biol. Interact..

[56] El-Khoury V., Moussay E., Janji B., Palissot V., Aouali N., Brons N.H., Van Moer K., Pierson S., Van Dyck E., Berchem G. (2010). The histone deacetylase inhibitor MGCD0103 induces apoptosis in B-cell chronic lymphocytic leukemia cells through a mitochondria-mediated caspase activation cascade. Mol. Cancer Ther..

[57] Rigobello M.P., Scutari G., Boscolo R., Bindoli A. (2002). Induction of mitochondrial permeability transition by auranofin, a gold(I)-phosphine derivative. Br. J. Pharmacol..

[58] Zou P., Chen M., Ji J., Chen W., Chen X., Ying S., Zhang J., Zhang Z., Liu Z., Yang S., Liang G. (2015). Auranofin induces apoptosis by ROS-mediated ER stress and mitochondrial dysfunction and displayed synergistic lethality with piperlongumine in gastric cancer. Oncotarget.

[59] Bergquist P.L. (1962). Effect of 8-azaguanine on oxidative phosphorylation of mouse-liver mitochondria. Biochim. Biophys. Acta.

[60] Soltoff S.P. (2001). Rottlerin is a mitochondrial uncoupler that decreases cellular ATP levels and indirectly blocks protein kinase Cdelta tyrosine phosphorylation. J. Biol. Chem..

[61] Soltoff S.P. (2007). Rottlerin: An inappropriate and ineffective inhibitor of PKCdelta. Trends Pharmacol. Sci..

[62] Cammer W., Moore C.L. (1972). The effect of hexachlorophene on the respiration of brain and liver mitochondria. Biochem. Biophys. Res. Commun..

[63] Oliver C.L., Miranda M.B., Shangary S., Land S., Wang S., Johnson D.E. (2005). (-)-Gossypol acts directly on the mitochondria to overcome Bcl-2- and Bcl-X(L)-mediated apoptosis resistance. Mol. Cancer Ther..

[64] Warnsmann V., Meyer N., Hamann A., Kögel D., Osiewacz H.D. (2018). A novel role of the mitochondrial permeability transition pore in (-)-gossypol-induced mitochondrial dysfunction. Mech. Ageing Dev..

[65] Tu P., Gibon J., Bouron A. (2010). The TRPC6 channel activator hyperforin induces the release of zinc and calcium from mitochondria. J. Neurochem..

[66] Schempp C.M., Kirkin V., Simon-Haarhaus B., Kersten A., Kiss J., Termeer C.C., Gilb B., Kaufmann T., Borner C., Sleeman J.P., Simon J.C. (2002). Inhibition of tumour cell growth by hyperforin, a novel anticancer drug from St. John's wort that acts by induction of apoptosis. Oncogene.

[67] Tao H., Zhang Y., Zeng X., Shulman G.I., Jin S. (2014). Niclosamide ethanolamine-induced mild mitochondrial uncoupling improves diabetic symptoms in mice. Nat. Med..

[68] Park S.J., Shin J.H., Kang H., Hwang J.J., Cho D.H. (2011). Niclosamide induces mitochondria fragmentation and promotes both apoptotic and autophagic cell death. BMB Rep..

[69] Wan K.F., Chan S.L., Sukumaran S.K., Lee M.C., Yu V.C. (2008). Chelerythrine induces apoptosis through a Bax/Bak-independent mitochondrial mechanism. J. Biol. Chem..

[70] Zhang Z.F., Guo Y., Zhang J.B., Wei X.H. (2011). Induction of apoptosis by chelerythrine chloride through mitochondrial pathway and Bcl-2 family proteins in human hepatoma SMMC-7721 cell. Arch. Pharm. Res..

[71] Zhao X., Cai A., Peng Z., Liang W., Xi H., Li P., Chen G., Yu J., Chen L. (2019). JS-K induces reactive oxygen species-dependent anti-cancer effects by targeting mitochondria respiratory chain complexes in gastric cancer. J. Cell. Mol. Med..

[72] Tan W., Zhong Z., Wang S., Suo Z., Yang X., Hu X., Wang Y. (2015). Berberine regulated lipid metabolism in the presence of C75, compound C, and TOFA in breast cancer cell line MCF-7. Evid. Based Complement. Alternat. Med..

[73] Khan R., Schmidt-Mende J., Karimi M., Gogvadze V., Hassan M., Ekström T.J., Zhivotovsky B., Hellström-Lindberg E. (2008). Hypomethylation and apoptosis in 5-azacytidine-treated myeloid cells. Exp. Hematol..

[74] Cosimo E., McCaig A.M., Carter-Brzezinski L.J., Wheadon H., Leach M.T., Le Ster K., Berthou C., Durieu E., Oumata N., Galons H., Meijer L., Michie A.M. (2013). Inhibition of NF-κB-mediated signaling by the cyclin-dependent kinase inhibitor CR8 overcomes prosurvival stimuli to induce apoptosis in chronic lymphocytic leukemia cells. Clin. Cancer Res..

[75] Wang Y., Biswas G., Prabu S.K., Avadhani N.G. (2006). Modulation of mitochondrial metabolic function by phorbol 12-myristate 13-acetate through increased mitochondrial translocation of protein kinase Calpha in C2C12 myocytes. Biochem. Pharmacol..

[76] Bensassi F., Gallerne C., Sharaf El Dein O., Lemaire C., Hajlaoui M.R., Bacha H. (2012). Involvement of mitochondria-mediated apoptosis in deoxynivalenol cytotoxicity. Food Chem. Toxicol..

[77] Ma Y., Zhang A., Shi Z., He C., Ding J., Wang X., Ma J., Zhang H. (2012). A mitochondria-mediated apoptotic pathway induced by deoxynivalenol in human colon cancer cells. Toxicol. In Vitro.

[78] Cao Y., Wang J., Tian H., Fu G.H. (2020). Mitochondrial ROS accumulation inhibiting JAK2/STAT3 pathway is a critical modulator of CYT997-induced autophagy and apoptosis in gastric cancer. J. Exp. Clin. Cancer Res..

[79] Hom J.R., Gewandter J.S., Michael L., Sheu S.S., Yoon Y. (2007). Thapsigargin induces biphasic fragmentation of mitochondria through calcium-mediated mitochondrial fission and apoptosis. J. Cell Physiol..

[80] Vercesi A.E., Moreno S.N., Bernardes C.F., Meinicke A.R., Fernandes E.C., Docampo R. (1993). Thapsigargin causes Ca2+ release and collapse of the membrane potential of Trypanosoma brucei mitochondria *in situ* and of isolated rat liver mitochondria. J. Biol. Chem..

[81] Sen S., Sharma H., Singh N. (2005). Curcumin enhances Vinorelbine mediated apoptosis in NSCLC cells by the mitochondrial pathway. Biochem. Biophys. Res. Commun..

[82] Yamada T., Egashira N., Imuta M., Yano T., Yamauchi Y., Watanabe H., Oishi R. (2010). Role of oxidative stress in vinorelbine-induced vascular endothelial cell injury. Free Radic. Biol. Med..

[83] Horrum M.A., Jennett R.B., Ecklund R.E., Tobin R.B. (1986). Inhibition of respiration in mitochondria and in digitonin-treated rat hepatocytes by podophyllotoxin. Mol. Cell. Biochem..

[84] Varalda M., Antona A., Bettio V., Roy K., Vachamaram A., Yellenki V., Massarotti A., Baldanzi G., Capello D. (2020). Psychotropic drugs show anticancer activity by disrupting mitochondrial and lysosomal function. Front. Oncol..

