# Supplementary material for: Labeling and measuring stressed mitochondria using a PINK1-based ratiometric fluorescent sensor
Source: J Biol Chem. 2021 Oct 5;297(5):101279. doi: 10.1016/j.jbc.2021.101279 (PMC8560995; doi:10.1016/j.jbc.2021.101279)
Supplement: Supplemental Figures S1–S3 and Tables S1, S2 [file mmc1.pdf]

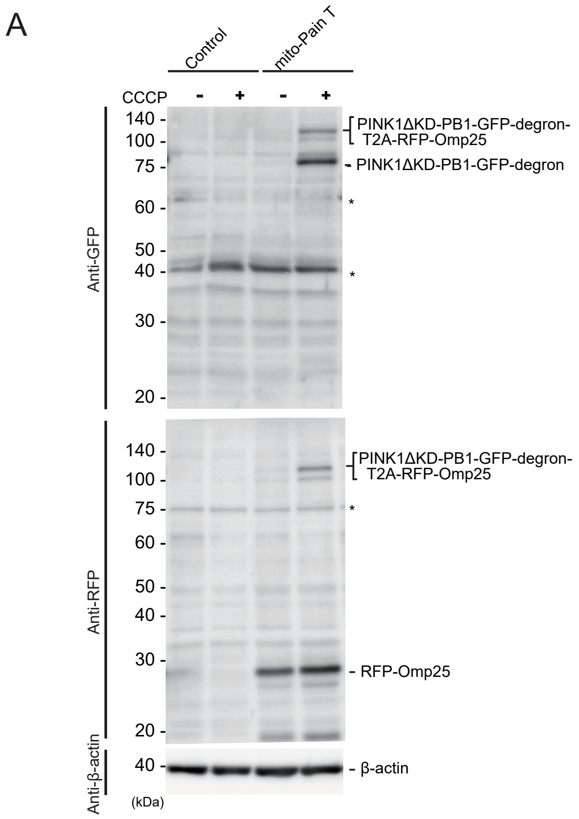

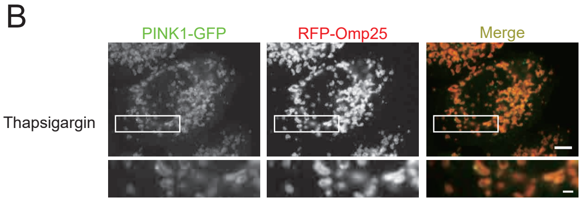

Figure S1. Analysis of mito-Pain T

A, Cleavage rate of T2A. HeLa cells expressing mito-Pain T were cultured with CCCP or DMSO for 24 h and then analyzed by immunoblotting using antibodies against GFP and RFP. *B*, Thapsigargin induced PINK1 localization to mitochondria. HeLa cells stably expressing mito-Pain T were cultured with thapsigargin for 24 h before fixation and observed by fluorescence microscopy. Scale bar, 5 µm. Inset scale bar, 1 µm.

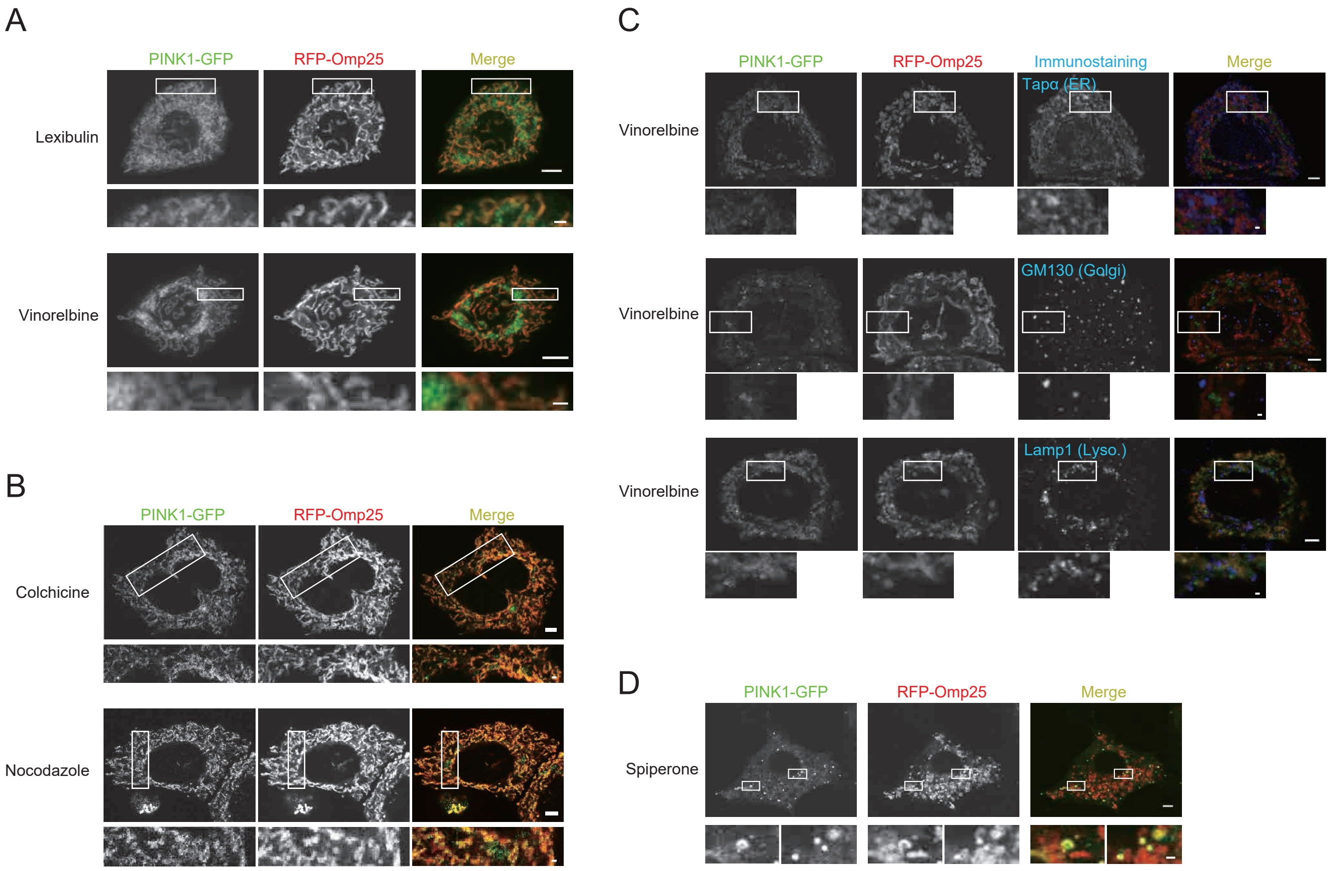

Figure S2. Ectopic PINK1-GFP localization

A, PINK1 -GFP localized not only mitochondria but also to unknown structures after lexibulin or vinorelbine treatment. HeLa cells stably expressing mito-Pain T were cultured with the indicated compounds for 24 h before fixation and observed by fluorescence microscopy. Scale bar, 5 µm. Inset scale bar, 1 µm. *B*, Some inhibitors of tubulin polymerization induced ectopic PINK1 localization. HeLa cells stably expressing mito-Pain F were cultured with the indicated compounds for 24 h before fixation and observed by fluorescence microscopy Scale bar, 5 µm. Inset scale bar, 1 µm. *C.* Ectopic PINK1 didn't colocalize with microtubule-organizing organelles. HeLa cells stably expressing mito-Pain F were cultured with vinorelbine for 24 h before fixation. Cells were stained with antibodies against Trapα, GM130 or Lamp1 and observed by fluorescence microscopy. Scale bar, 5 µm. Inset scale bar, 1 µm. *D.* Spiperone induced PINK1-GFP puncta to partially colocalize with mitochondria. COS cells stably expressing mito-Pain F were cultured with spiperone for 24 h before fixation and observed by fluorescence microscopy. Scale bar, 5 µm. Inset scale bar, 1 µm.

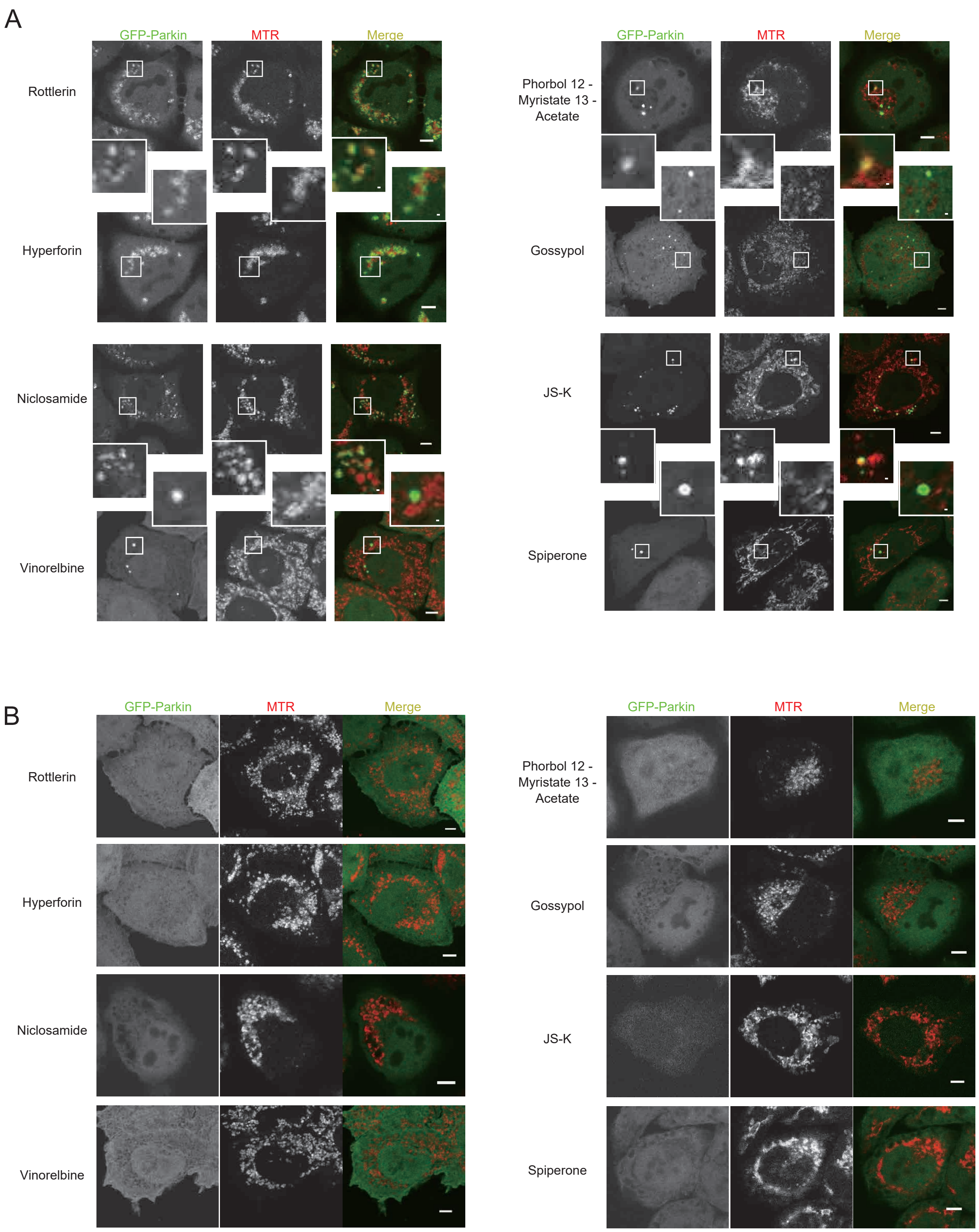

Figure S3. Ectopic GFP-Parkin localization

A. Some compounds led to Parkin redistribution. HeLa cells expressing GFP-Parkin were cultured with rottlerin, hyperforin, niclosamide, vinorelbine or PMA for 6 h and gossypol, JS-K, or spiperone for 24 h before fixation and observed by fluorescence microscopy. Scale bar, 5 µm. Inset scale bar, 1 µm. *B*. PINK1 was essential for Parkin recruitment by hit compounds. PINK1-KO HeLa cells expressing GFP-Parkin were cultured with rottlerin, hyperforin, niclosamide, vinorelbine or PMA for 6 h and by gossypol, JS-K or spiperone for 24 h before fixation and observed by fluorescence microscopy. Scale bar, 5 µm.

Ranking	Drug name	PINK1 stability	function
1	MG-115*	39.46	20S and 26S proteasome inhibitor
2	Apicidin*	35.14	HDAC inhibitor
3	Hellebrin*	24.42	Na^{+}/K^{+}-ATPase inhibitor
4	Mocetinostat	14.75	HDAC1 inhibitor
5	β-Rubromycin	8.28	TERT
6	Auranofin	8.12	thioredoxin reductase (TrxR) inhibitor
7	Azaguanine-8	6.39	purine analogue and compete with guanine
8	Rottlerin	5.75	PKC and CaM kinase III inhibitor
9	Linifanib	5.06	receptor tyrosine kinase inhibitor
10	Nonactin	4.72	ionophore
11	Hexachlorophene	4.52	KCNQ1/KCNE1 potassium channel activator
12	Gossypol	4.50	Bcl-XL, Bcl-2 and Mcl-1 inhibitor
13	Hyperforin	4.49	SIRT1 inhibitor
14	Niclosamide	4.04	STAT3 inhibitor
15	Chelerythrine*	3.83	Bcl-XL-Bak binding inhibitor
16	JS-K	3.55	generate NO
17	ROPA	3.31	PKC activator
18	TOFA	3.07	acetyl-coenzyme A carboxylase inhibitor
19	Lycorine	3.05	Peptidyl transferase center (PTC) inhibitor
20	Cryptotanshinone	2.89	STAT3 inhibitor
21	5-azacytidine	2.76	DNA methyltransferase inhibitor
22	(R)-CR8	2.74	CDK1, 2, 5, 7 and 9 inhibitor
23	Rac-2-Ethoxy-3-hexadecanamido-1-propylphosphocholine	2.38	PKC inhibitor
24	Hexetidine	2.32	inhibitor of growth of bacteria
25	Regorafenib	2.28	tyrosine kinase inhibitor
26	T0901317	2.21	liver X receptor (LXR) agonist
27	SU 9516	2.12	Cdk2 inhibitor
28	Phorbol 12-Myristate 13-Acetate	2.11	PKC activator
29	Mercaptopurine	2.07	purine systhesis inhibitor
30	BAY 43-9006	2.05	RAF/MEK/ERK and tyrosine kinases inhibitor
31	Clofilium	1.95	K^{+} channel blocker
32	Diphenyleneiodonium	1.82	nitric oxide synthase inhibitor
33	PD-407824	1.74	Wee1/Chk1 inhibitor
34	Deoxynivalenol*	1.70	bind to ribosome and inhibit protein synthesis
35	ARP 101	1.70	matrix metalloproteinase 2 inhibitor
36	Lexibulin	1.65	tubulin polymerization inhibitor
37	Tubulozole	1.63	microtubule inhibitor
38	Adapalene	1.62	RARβ and RARγ agonist
39	Securinine	1.61	GABA receptor antagonist
40	Thapsigargin	1.56	ER Ca^{2+}-ATPases
41	Tomatine	1.54	NF-κB Akt inhibitor
42	Cyclopiazonic Acid	1.48	Ca^{2+}-ATPases inhibitor
43	Methiazole	1.41	anthelmintic
44	Vinorelbine	1.39	tubulin polymerization inhibitor
45	Telaprevir	1.38	HCV NS3-4A serine protease inhibitor
46	Podophyllotoxin	1.38	tubulin polymerization inhibitor
47	CP-31398	1.36	p53 activator
48	Pifithrin-μ	1.35	reduce an affinity of p53 to Bcl-xL and Bcl-2
49	Piperlongumine	1.34	TrxR1 inhibitor
50	Albendazole	1.31	tubulin polymerization inhibitor
51	Rigosertib	1.30	multi-kinase inhibitor
52	Naloxonazine	1.29	mu1 opioid receptor antagonist
53	Luminespib	1.29	HSP90 inhibitor
54	SKF 96365	1.29	TRPC channels inhibitor
55	KX2-391	1.28	Src (tyrosine-protein kinase) inhibitor
56	Spiperone	1.27	Ca^{2+} activated Cl^{-}channels activator
57	PF-573228	1.27	focal adhesion kinase (FAC) inhibitor

Table S1 Hit compounds identified by screening.

Cells were treated with each compound for 24 h. Asterisk indicates toxic compounds, which induces cell death for 24 h treatment, were treated for 5 h.

**Drug name**	**Conc. (μM)**	**Source**	**Catalog Number**
MG-115	10	adipogen life sciences	AG-CP3-0015-M001
Apicidin	10	adipogen life sciences	AG-CN2-0087-M001
Hellebrin	10	cayman chemical	21442
Mocetinostat	10	cayman chemical	18287
β-Rubromycin	10	adipogen life sciences	BVT-0251-M001
Auranofin	10	adipogen life sciences	AG-CR1-3611-M025
Azaguanine-8	10	santa cruz biotechnology	sc-207194
Rottlerin	10	adipogen life sciences	AG-CN2-0526-M010
Linifanib	10	Toronto Research Chemicals	L466450
Hexachlorophene	10	cayman chemical	23948
Gossypol	10	LKT LABS	G5874
Hyperforin	10	adipogen life sciences	AG-CN2-0008-C500
Niclosamide	10	adipogen life sciences	AG-CR1-3643-M100
Chelerythrine	10	adipogen life sciences	AG-CR1-0071-M001
JS-K	10	cayman chemical	21225
TOFA	10	cayman chemical	10005263
(R)-CR8	10	adipogen life sciences	AG-CR1-0039-M001
Lexibulin	10	cayman chemical	18395
Vinorelbine	10	LKT LABS	V3252
Podophyllotoxin	10	cayman chemical	19575
Spiperone	10	Toronto Research Chemicals	S682000
Chloroquine	20	Wako	038-17971
Bafilomycin A_1	0.1	LC lab	B-1080
Torin 1	1	Tocris	4247
Thapsigargin	0.2	Santa cruz	sc-24017
CCCP	20	nacalai tesque	07253-74
Oligomycin A	1	Calbiochem	495455
Antimycin A	1	Sigma	A8674-25MG
Valinomycin	2	Wako	228-01121
MG-132	5	Life sensors	SI9710
5-azacytidine	10	cayman chemical	11164
Phorbol 12-myristate 13-acetate	10	cayman chemical	10008014
Deoxynivalenol	10	cayman chemical	11428
Brefeldin A	10 (μg/ml)	Wako	022-15991
Tunicamycin	2 (μg/ml)	Sigma	T7765-1MG
Pepstatin A	20 (μg/ml)	PEPTIDE INSTITUTE	4397
Nocodazole	5 (μg/ml)	cayman chemical	13857
Colchicine	5 (μg/ml)	Wako	039-03851

Table S2 Compound concentrations used in this study
